# Correction: Study on the change characteristics and market driving factors of residential land price in the Beijing Tianjin Hebei urban agglomeration, China

**DOI:** 10.1371/journal.pone.0347067

**Published:** 2026-04-13

**Authors:** The PLOS One Editors

## Notice of Republication

This article was republished on March 24, 2026, to correct an error in the title: the title of [1] was erroneously duplicated from the title of [2]. Please download this article again to view the correct version. The originally published, uncorrected article and the republished, corrected article are provided here for reference.

In addition, the *PLOS One* Editors noted that the earlier article [2] was not cited and discussed in [1]. During post-publication discussions, corresponding author CL provided the following additional information:

[1] and [2] both analyzed land prices in the Beijing-Tianjin-Hebei region of China. However, there are differences in their research methods, content, and conclusions, specifically:

- In [2], socio-economic, transportation, and comprehensive land price data for the Beijing-Tianjin-Hebei urban agglomeration in 2017 were used. Employing the gravity model and an improved Voronoi model, [2] analysed the characteristics of comprehensive land price gravity, the types of interactions, the gravity pattern, and the issues present in land market development. In [1], a more detailed analysis specifically focused on residential land prices in the Beijing-Tianjin-Hebei region was conducted. Based on land transaction data from 2014 to 2017 and utilising Exploratory Spatial Data Analysis (ESDA) and the Geographically Weighted Regression (GWR) model, [1] revealed the spatio-temporal evolution characteristics and driving mechanisms of residential land prices.
- [2] found that the land price gravity values for cities such as Beijing, Tianjin, Langfang, and Tangshan (11.24 ~ 63.35) were significantly higher than those for other cities (0 ~ 6.09). The gravity structure among cities surrounding Beijing and Tianjin was closely connected, while connections were looser among peripheral cities. Regarding the gravity pattern, the sphere of influence of urban land prices was not confined to the administrative boundaries of each city. The land price gravity value of the sub-urban agglomeration around Beijing accounted for 66.4% of the total value. A significant polarisation effect in land price levels and influence was evident in Beijing and Tianjin, whereas a locking effect was apparent in Xingtai and Handan in the southern part of the region.
- [1] found that residential land prices in Beijing and Tianjin were significantly higher than in other areas, while Zhangjiakou, Chengde, and the western mountainous areas recorded the lowest residential land prices. Over time, residential land prices gradually exhibited a multi-centre development trend, with locational correlation increasing. Influenced by the land finance model of local governments in China, factors such as the utilisation rate of stock land, revenue from residential land transfers, and the growth in residential land transaction areas significantly contributed to the rise in residential land prices. Conversely, under the combined influence of land market supply and demand mechanisms and government management, four indicators (the land supply rate, per capita residential land supply area, the degree of marketisation of residential land supply, and the frequency of residential land transactions) suppressed the increase in residential land prices. Regional differences existed in the overall impact of land market factors on residential land prices, with a stronger effect observed in the north-central parts of Beijing, Tianjin, and Hebei compared to the southern regions. This may have been related to the more active land markets and stricter macro-management policies in Beijing, Tianjin, and their surrounding areas.
- [1] and [2] are interconnected. [1] builds upon [2] by further analysing the change patterns of residential land prices in the Beijing-Tianjin-Hebei region from 2014 to 2017, with a specific focus on the driving role of land market factors.
- The 2014–2017 period was selected for [1] because it corresponds to the policy window from the proposal to initial implementation of the Beijing-Tianjin-Hebei Coordinated Development Strategy. The promulgation of the plan for the coordinated development of the Beijing-Tianjin-Hebei Region in 2015 marked the substantive advancement of this regional integration strategy. Therefore, 2014–2017 represents a starting point for observing how this national strategy influenced the regional land market. The objective of [1] was to document the initial response patterns of the residential land market under the impetus of the policy, establishing a baseline for subsequent studies.
- [1] presents the driving mechanisms and spatial differentiation patterns of residential land prices influenced by land market factors (such as land finance dependence, land supply structure, and degree of marketisation). These variable relationships elucidate what factors are driving land price changes and how the intensity of these factors differs across regions.

The *PLOS One* Editors also noted that the methods as described in [1] may not be reproducible. [1] states that residential land prices were obtained from the China Land Market Network, the websites of the Natural Resources Bureau of each city, and field research. Field research was not defined nor was a description of how it was conducted provided in [1]. During post-publication discussions, corresponding author CL provided the following clarifications:

The study in [1] involved conducting visits to the bureaus of natural resources and planning in several key cities and counties within the Beijing-Tianjin-Hebei region to:

- Identify and supplement missing data from the China Land Market Network and the websites of municipal natural resources bureau by cross-referencing with the internal records held by local authorities.
- Verifying transaction information (specific location, land grade) in public datasets for the 17, 800 land parcels used in the analysis in [1].
- Conduct interviews with local land management officials on the specific implementation details of land supply policies, the actual operational models of land finance, and local experiences with market regulation.

## Supporting information

S1 File Originally published, uncorrected article. (PDF)

S2 File Republished, corrected article. (PDF)
