# Supplementary figures and images for: Study on the change characteristics and market driving factors of residential land price in the Beijing Tianjin Hebei urban agglomeration, China
Source: PLoS One. 2021 Sep 1;16(9):e0256710. doi: 10.1371/journal.pone.0256710 (PMC8409656; doi:10.1371/journal.pone.0256710)

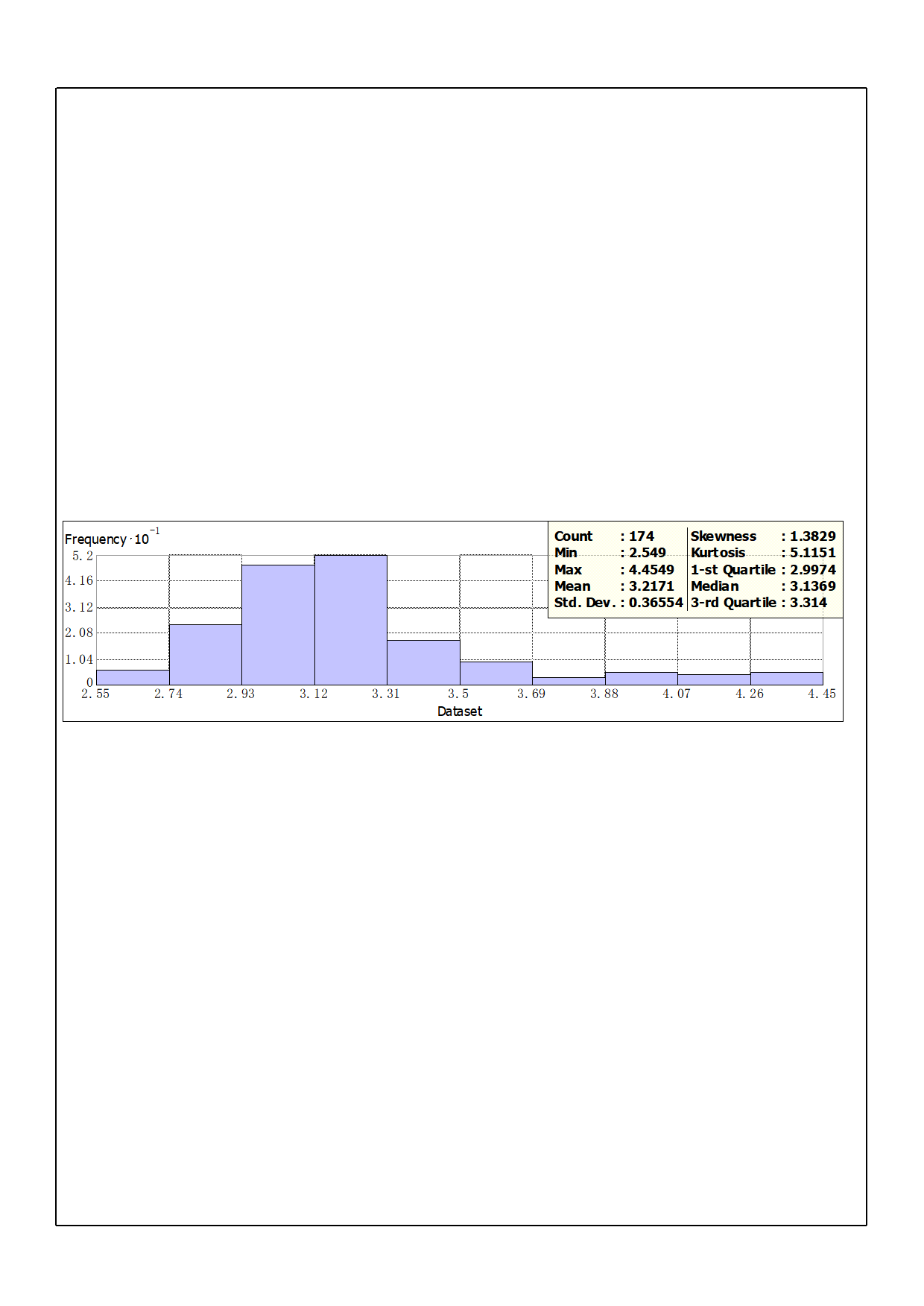

Supplement: S1 Fig — The normal distribution of residential land prices in 2014–2017. (ZIP) [file pone.0256710.s001.zip › 2014-Histogram .tif]

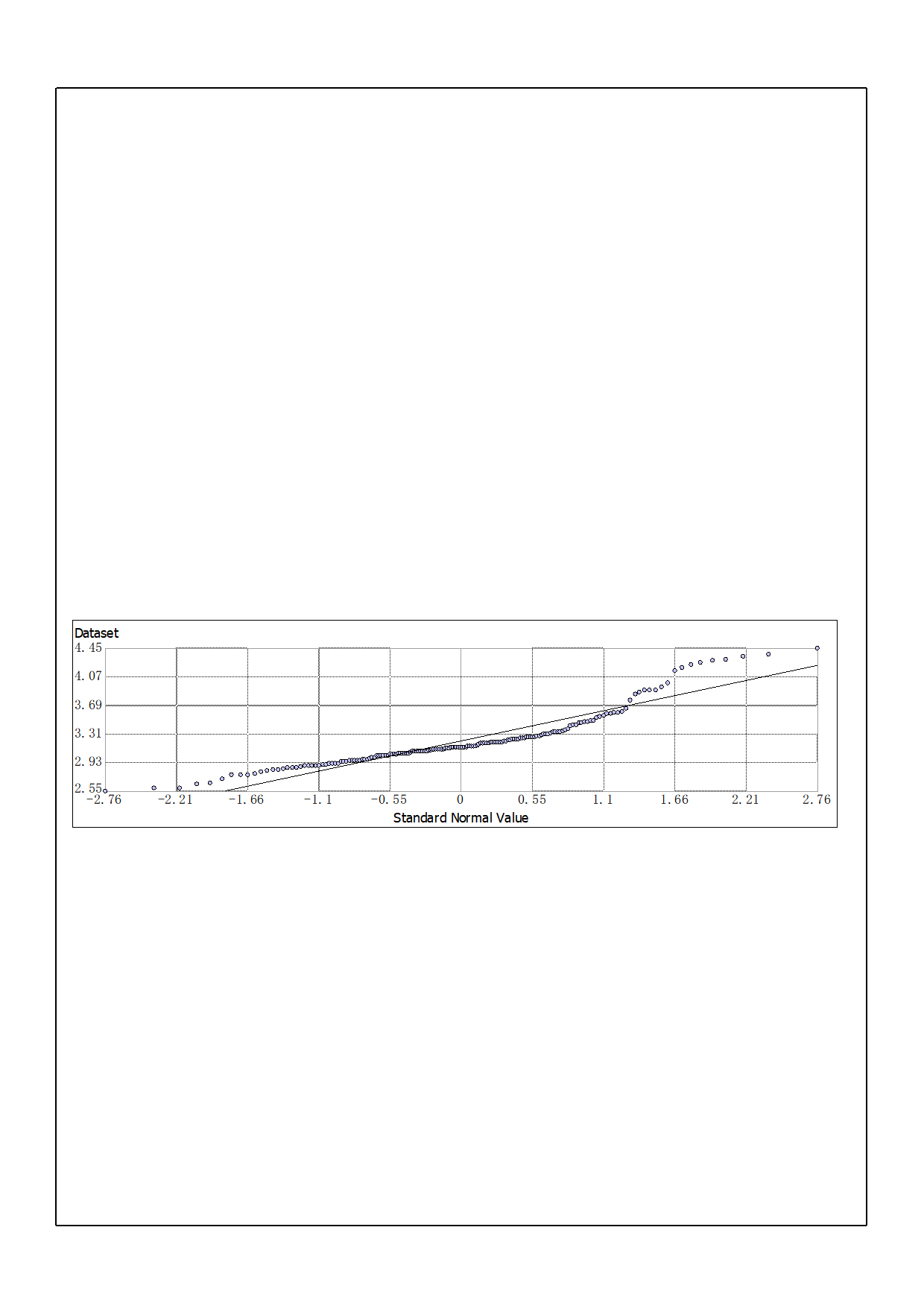

Supplement: S1 Fig — The normal distribution of residential land prices in 2014–2017. (ZIP) [file pone.0256710.s001.zip › 2014-QQ.tif]

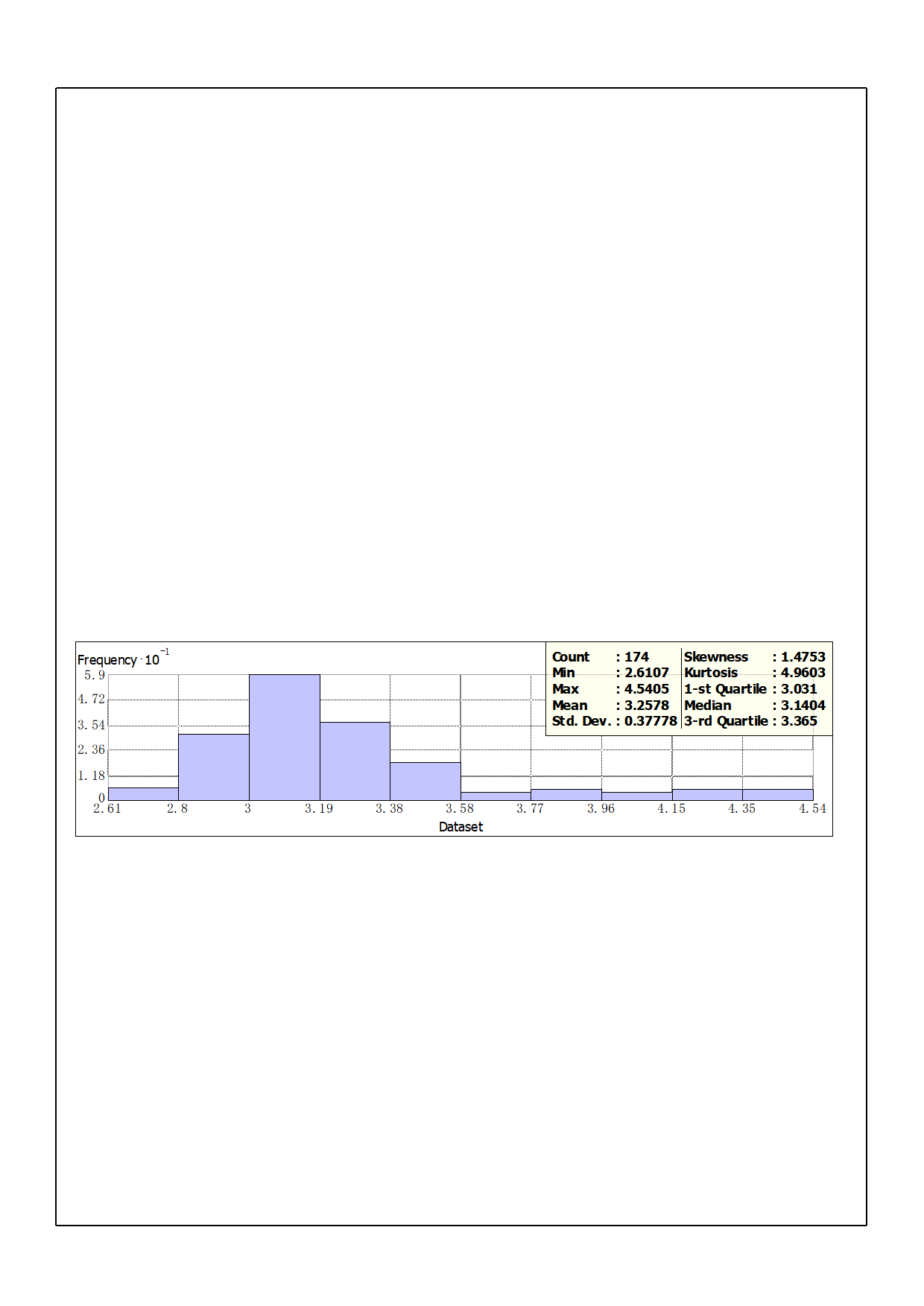

Supplement: S1 Fig — The normal distribution of residential land prices in 2014–2017. (ZIP) [file pone.0256710.s001.zip › 2015-Histogram .tif]

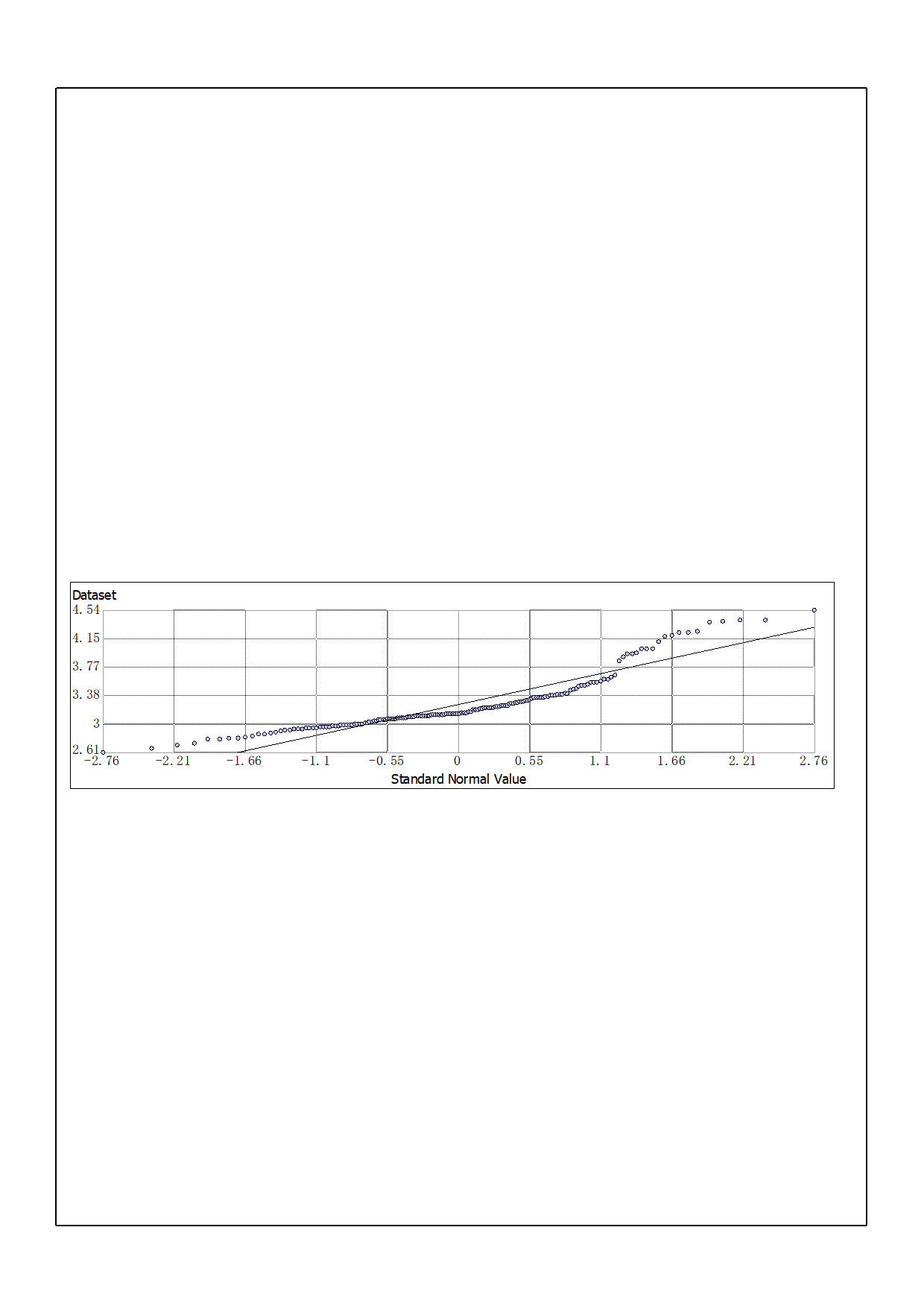

Supplement: S1 Fig — The normal distribution of residential land prices in 2014–2017. (ZIP) [file pone.0256710.s001.zip › 2015-QQ.tif]

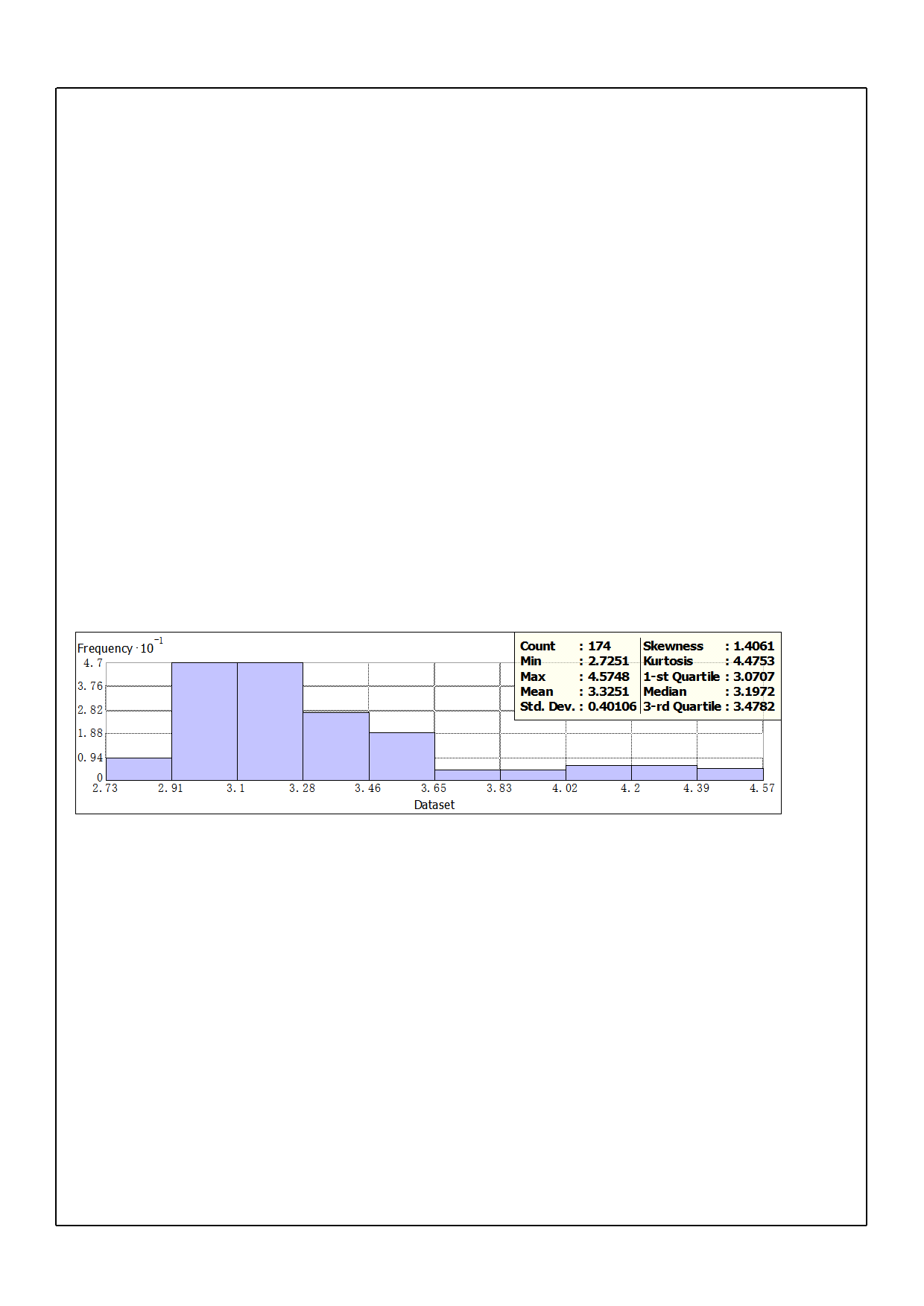

Supplement: S1 Fig — The normal distribution of residential land prices in 2014–2017. (ZIP) [file pone.0256710.s001.zip › 2016-Histogram .tif]

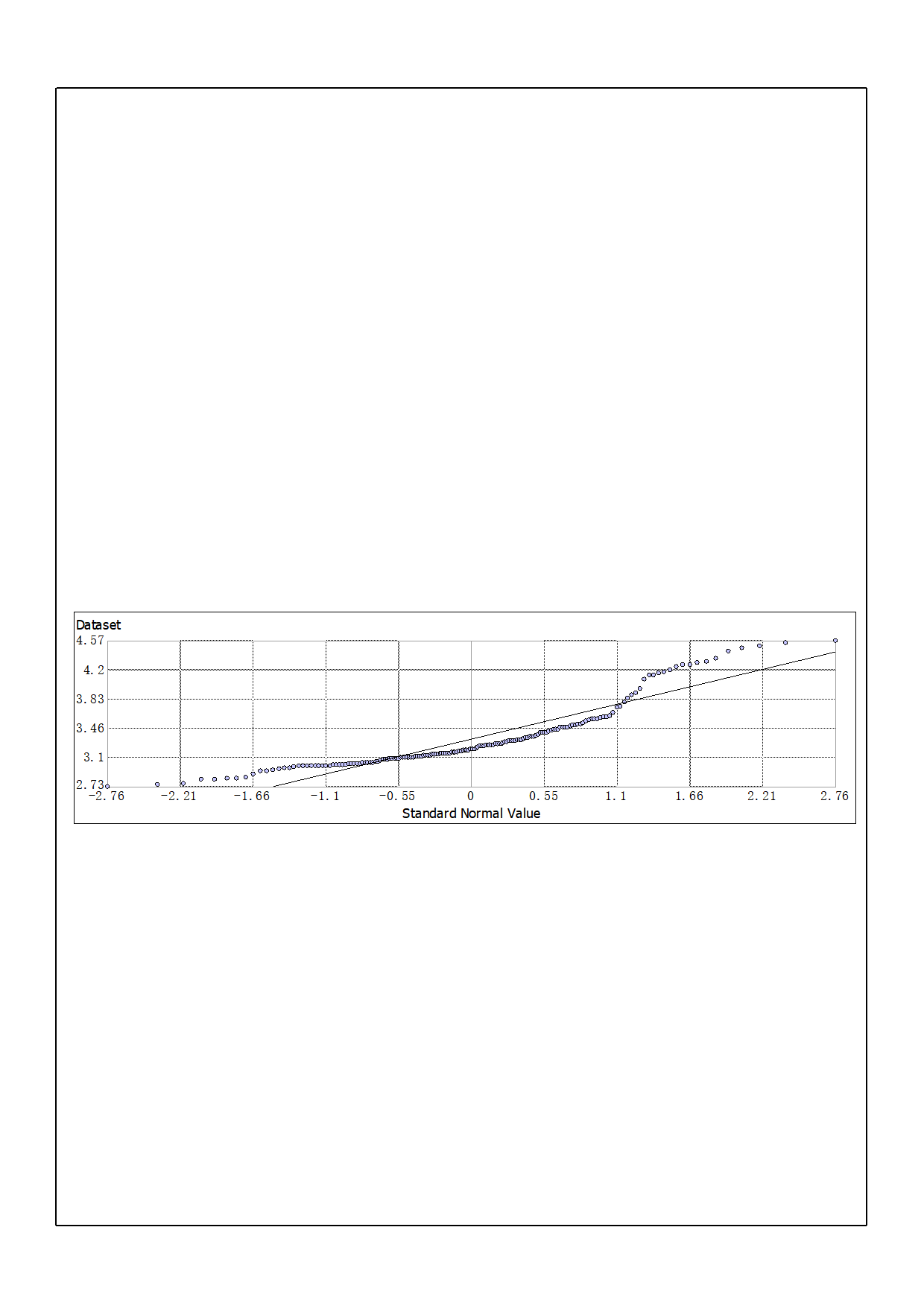

Supplement: S1 Fig — The normal distribution of residential land prices in 2014–2017. (ZIP) [file pone.0256710.s001.zip › 2016-QQ.tif]

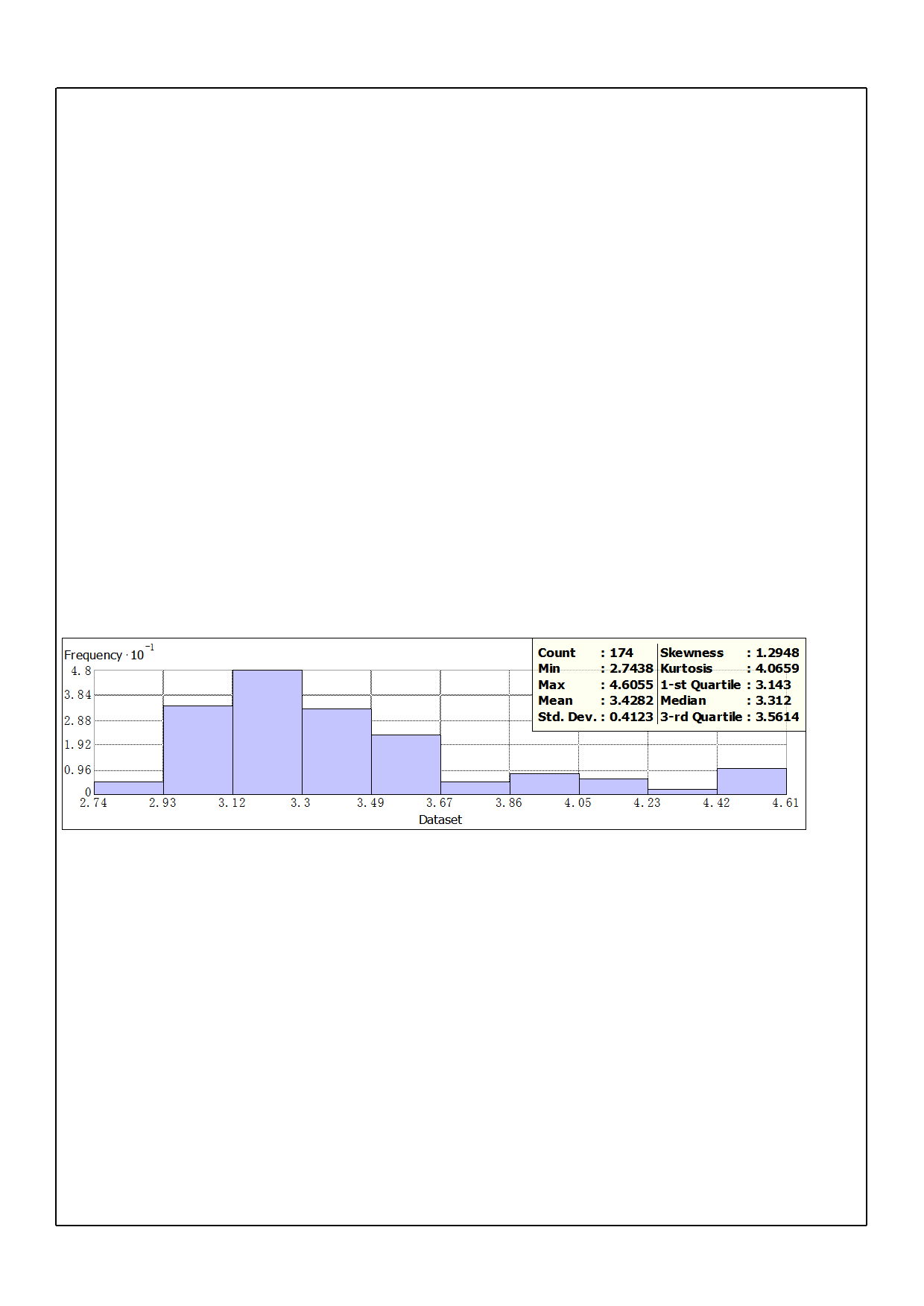

Supplement: S1 Fig — The normal distribution of residential land prices in 2014–2017. (ZIP) [file pone.0256710.s001.zip › 2017-Histogram .tif]

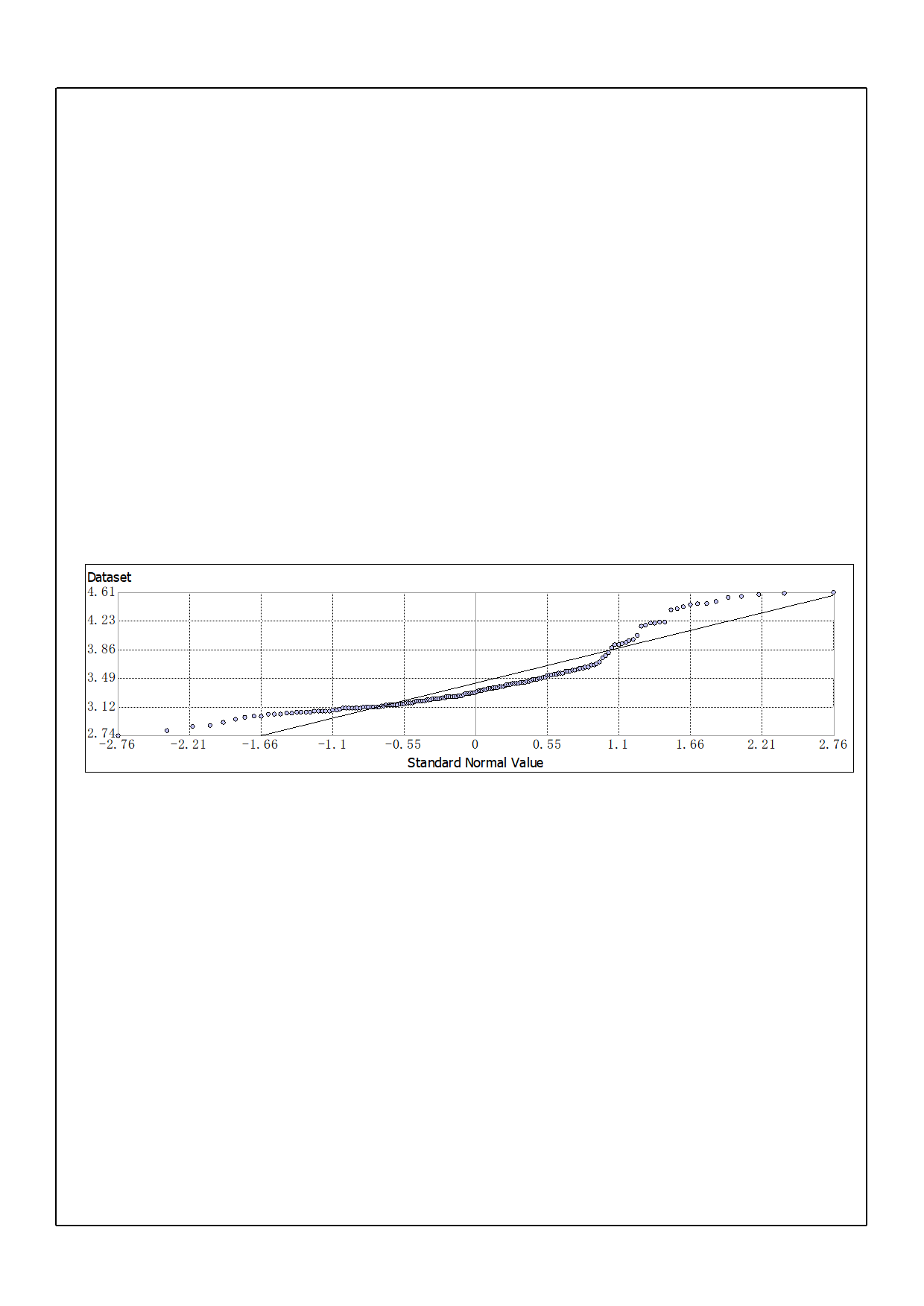

Supplement: S1 Fig — The normal distribution of residential land prices in 2014–2017. (ZIP) [file pone.0256710.s001.zip › 2017-QQ.tif]

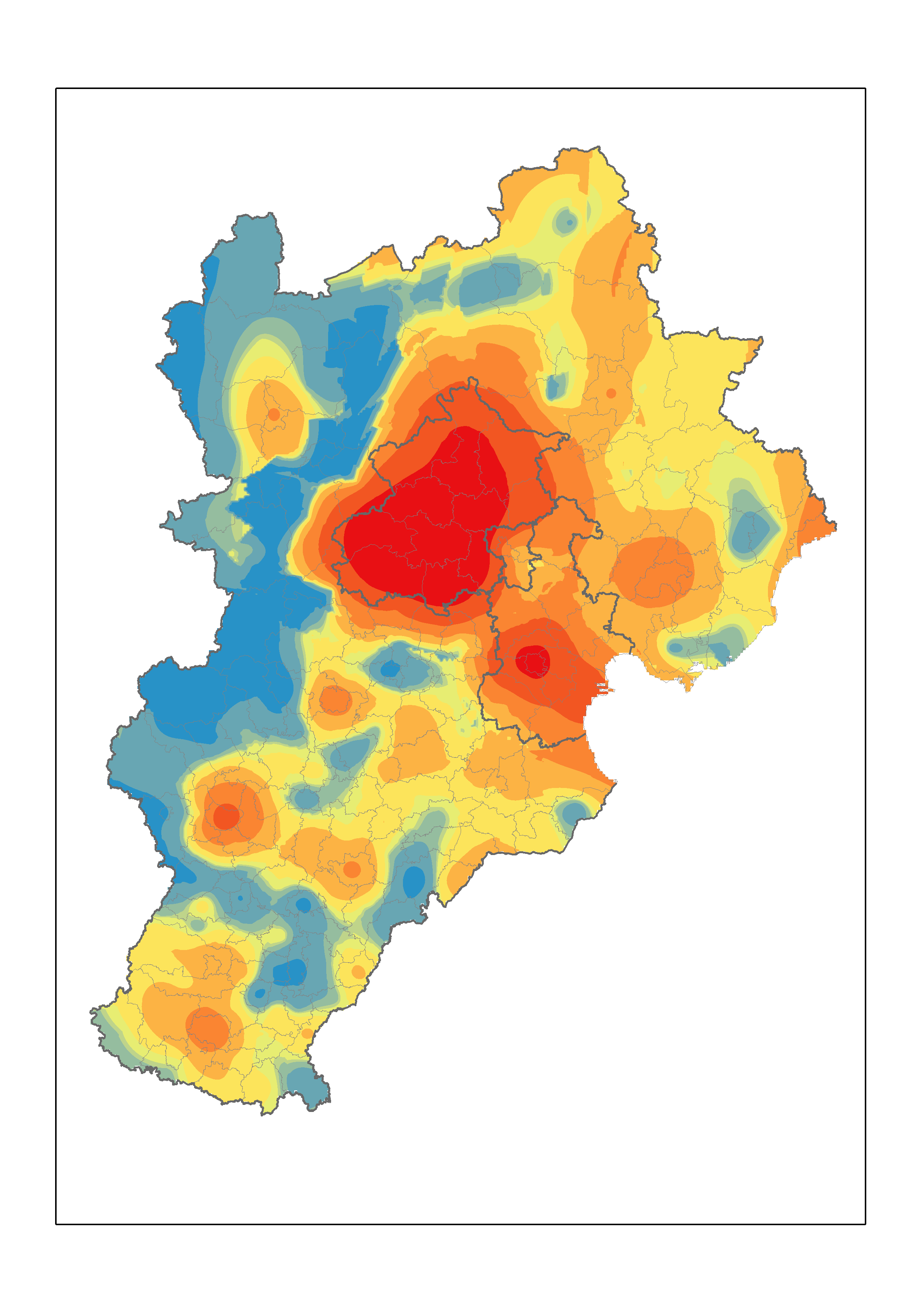

Supplement: S2 Fig — Kriging interpolation results from 2014–2017. (ZIP) [file pone.0256710.s002.zip › 2014Interpolation graph .tif]

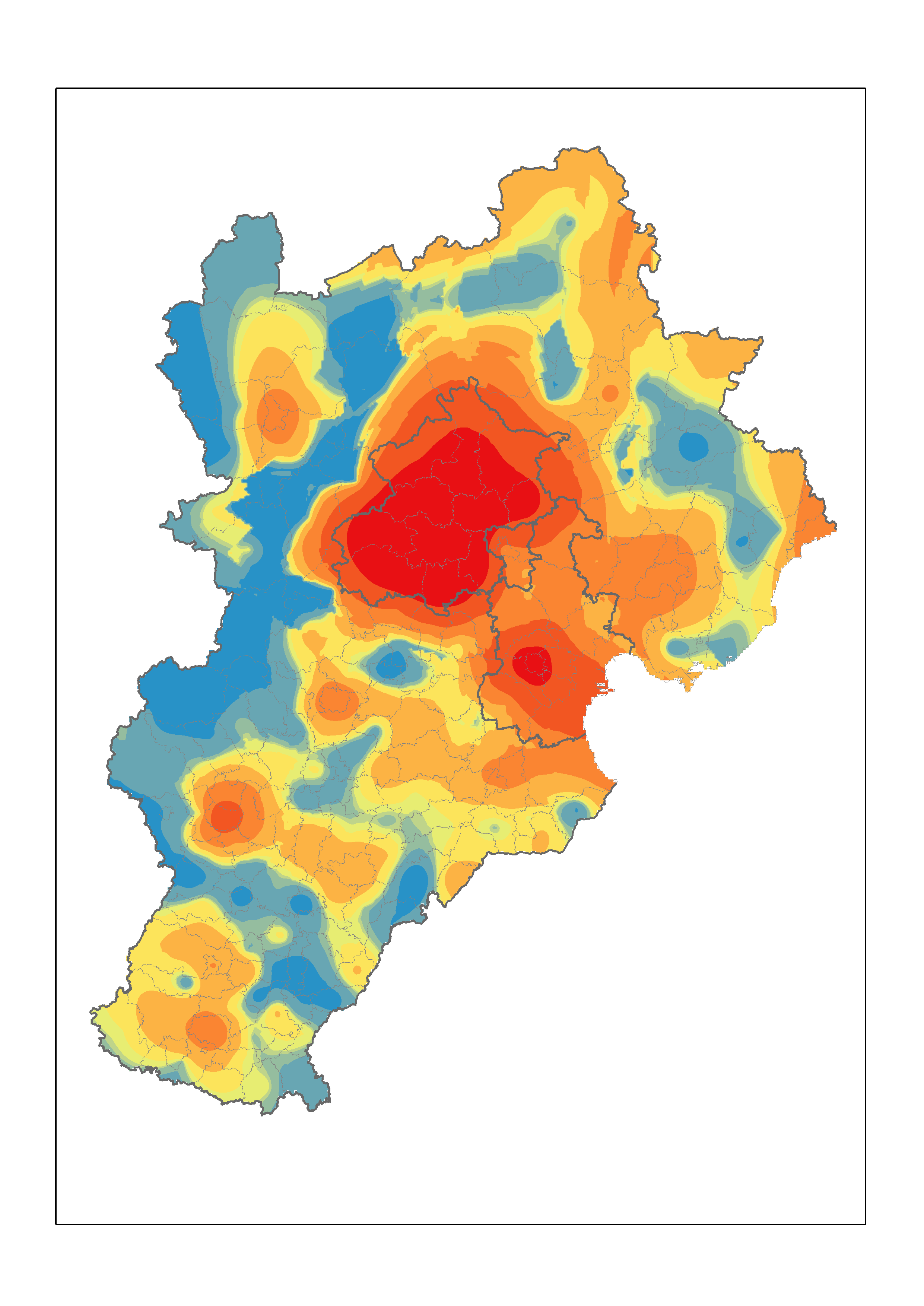

Supplement: S2 Fig — Kriging interpolation results from 2014–2017. (ZIP) [file pone.0256710.s002.zip › 2015Interpolation graph .tif]

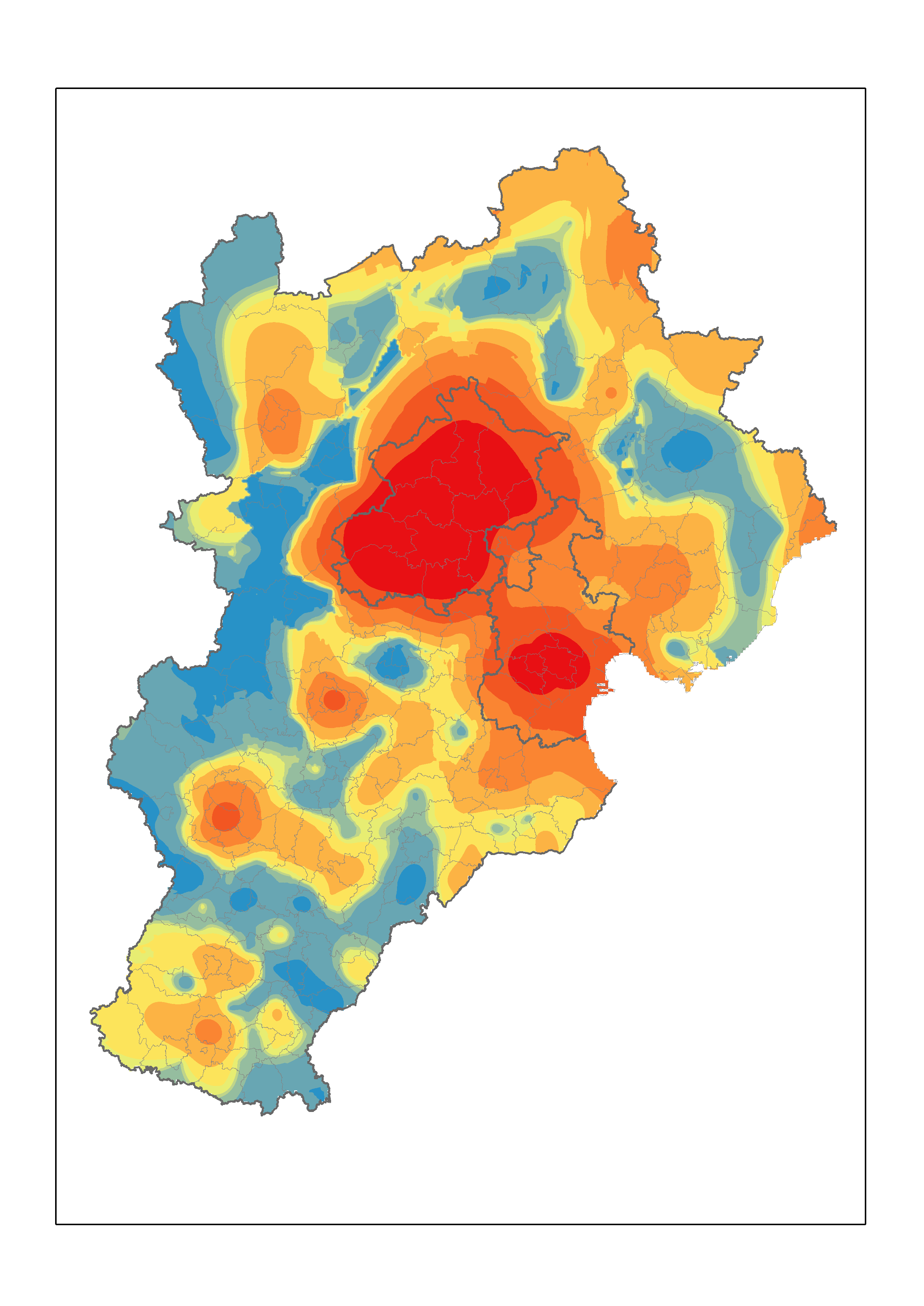

Supplement: S2 Fig — Kriging interpolation results from 2014–2017. (ZIP) [file pone.0256710.s002.zip › 2016Interpolation graph .tif]

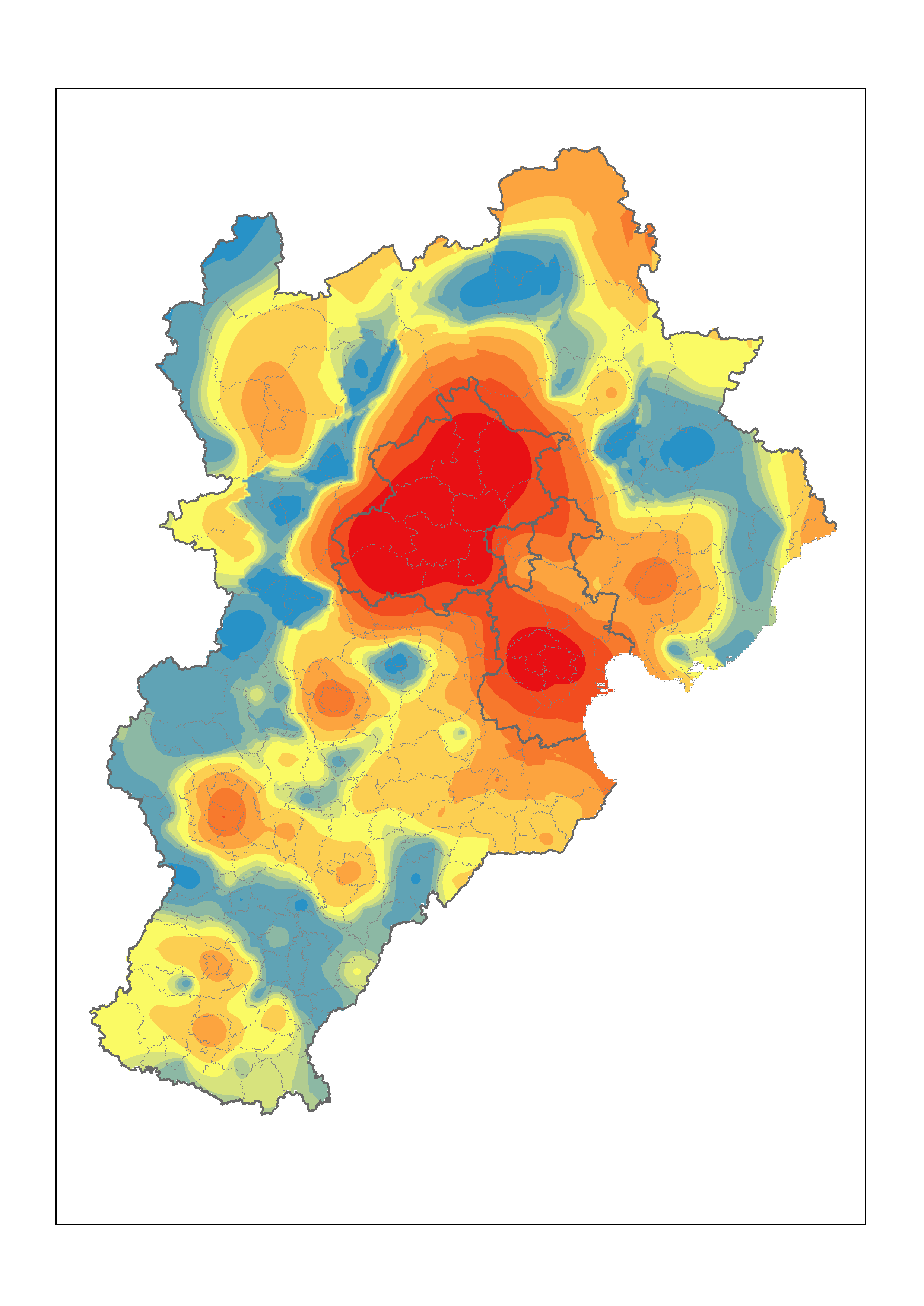

Supplement: S2 Fig — Kriging interpolation results from 2014–2017. (ZIP) [file pone.0256710.s002.zip › 2017Interpolation graph .tif]

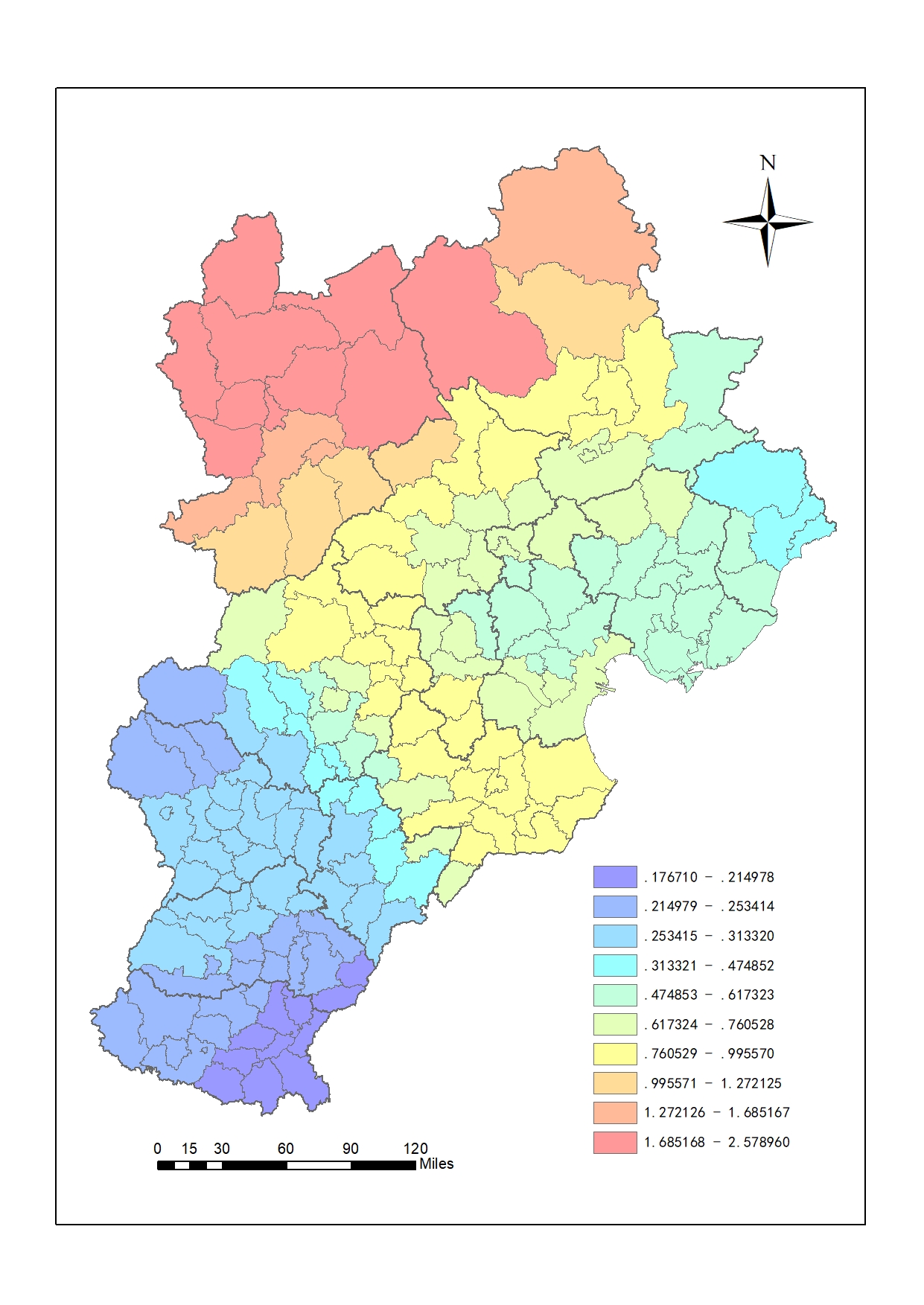

Supplement: S3 Fig — Distribution map of the driving effect of X4, X8, X10 on residential land prices. (ZIP) [file pone.0256710.s003.zip › X10.jpg]

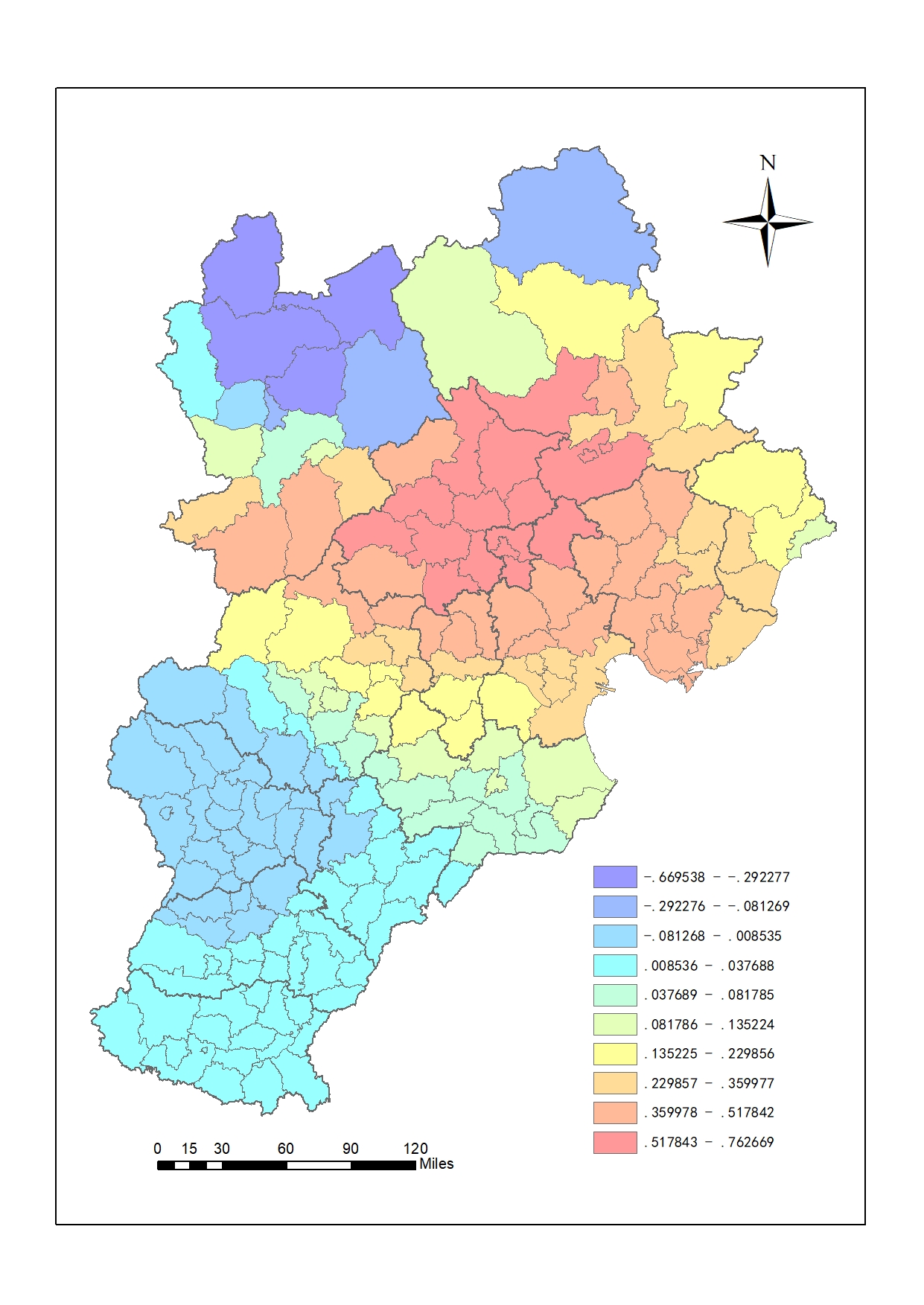

Supplement: S3 Fig — Distribution map of the driving effect of X4, X8, X10 on residential land prices. (ZIP) [file pone.0256710.s003.zip › X4.jpg]

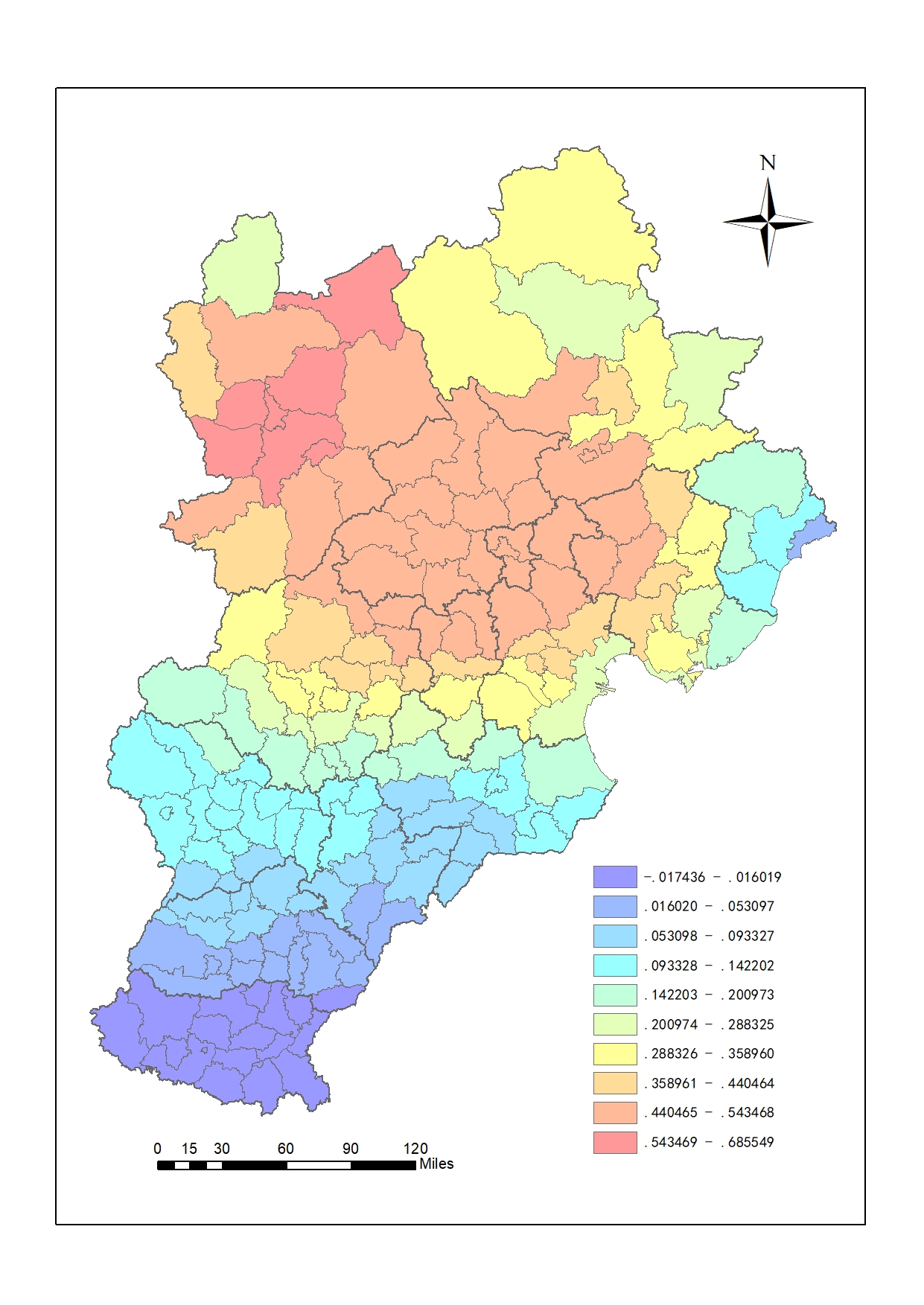

Supplement: S3 Fig — Distribution map of the driving effect of X4, X8, X10 on residential land prices. (ZIP) [file pone.0256710.s003.zip › X8.jpg]

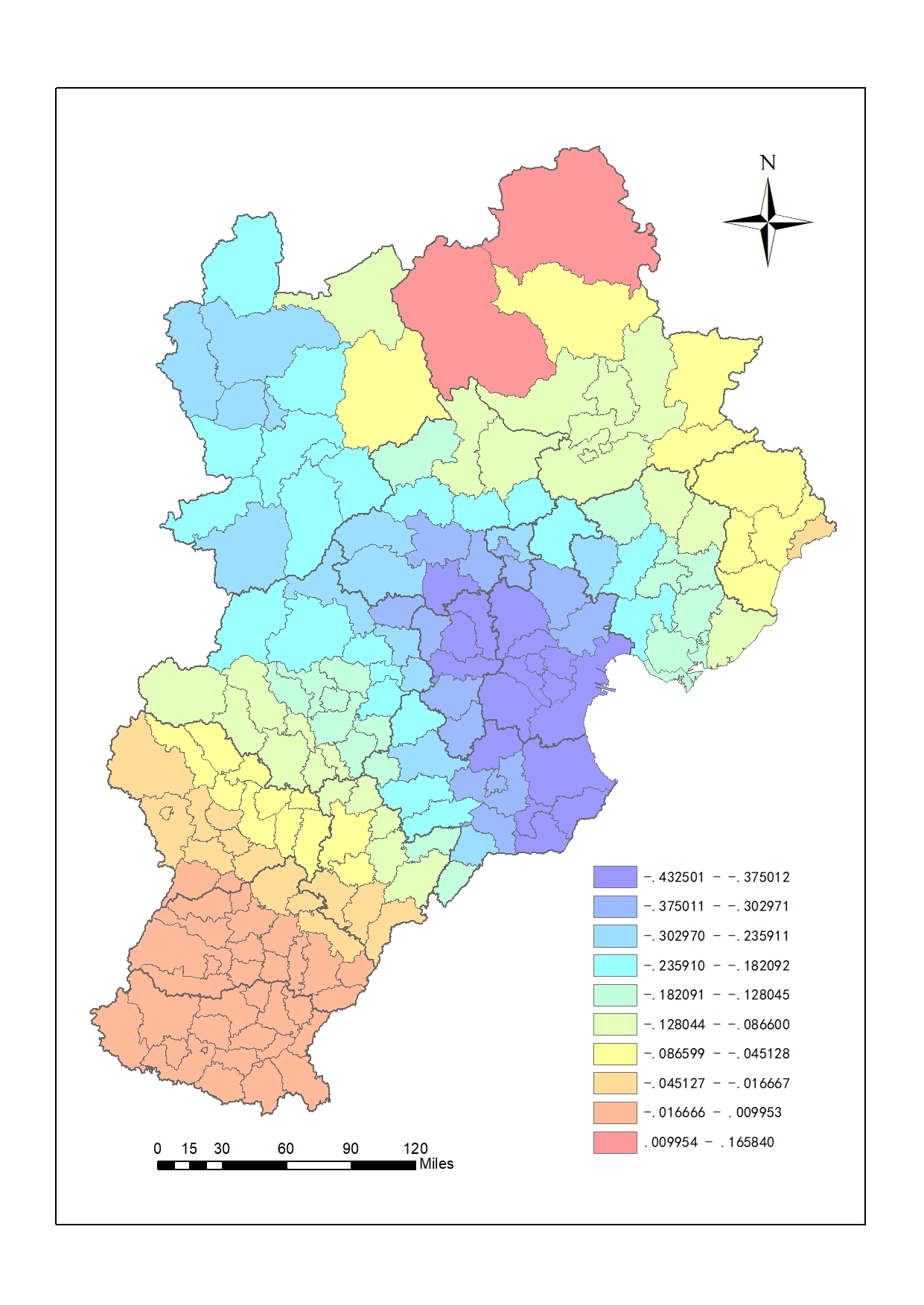

Supplement: S4 Fig — Distribution map of the driving effect of X3, X5, X6, X9 on residential land prices. (ZIP) [file pone.0256710.s004.zip › X3.jpg]

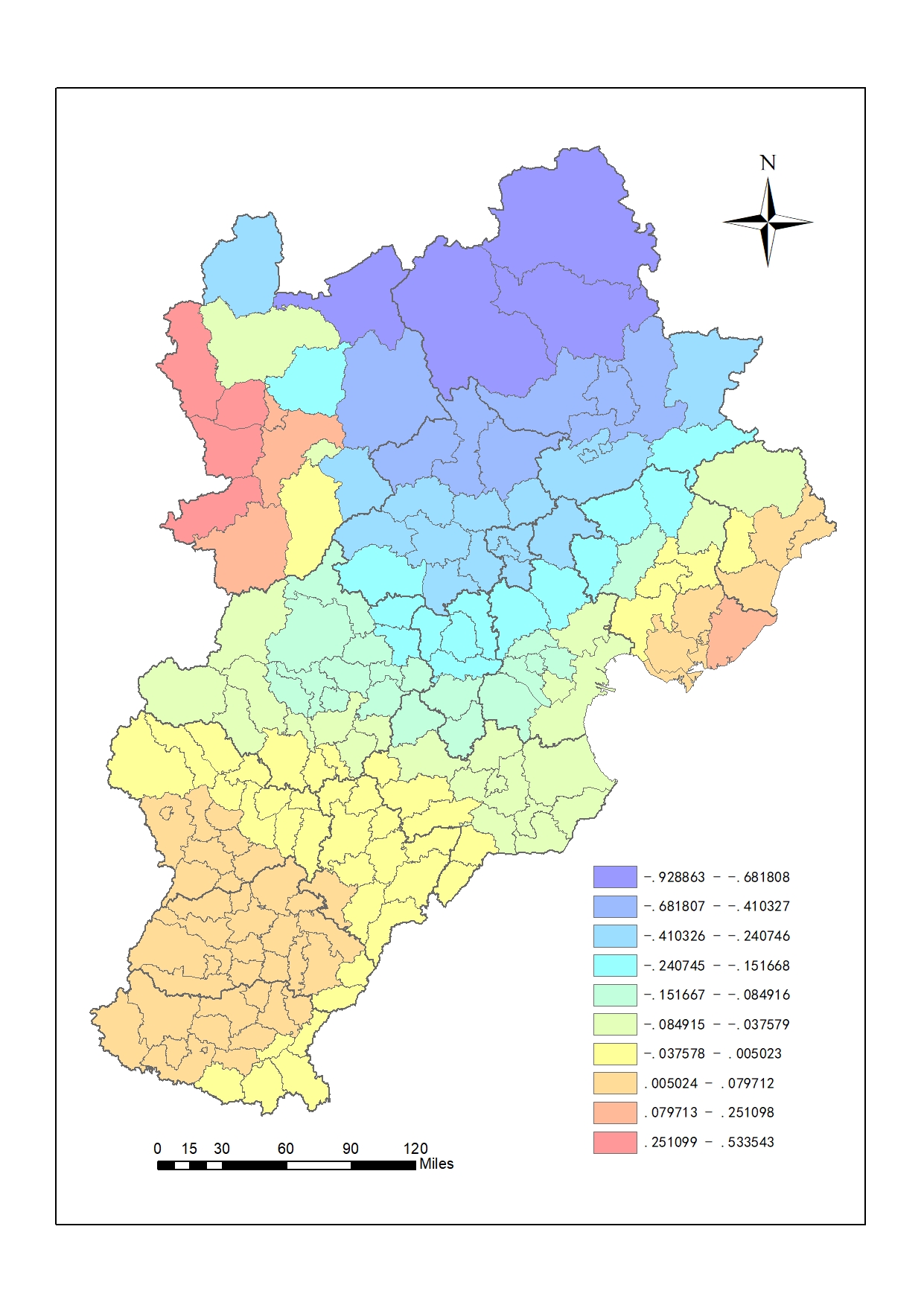

Supplement: S4 Fig — Distribution map of the driving effect of X3, X5, X6, X9 on residential land prices. (ZIP) [file pone.0256710.s004.zip › X5.jpg]

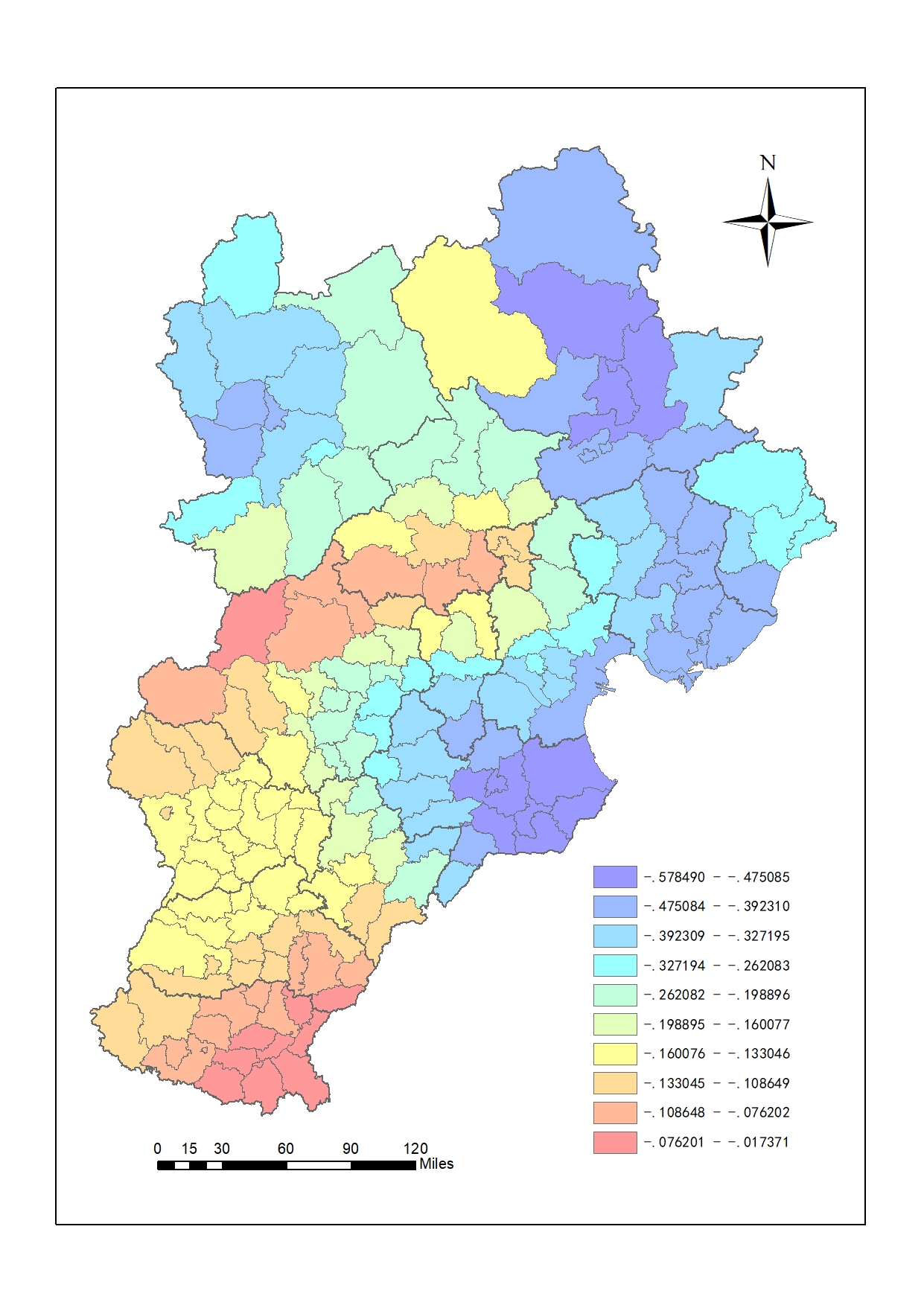

Supplement: S4 Fig — Distribution map of the driving effect of X3, X5, X6, X9 on residential land prices. (ZIP) [file pone.0256710.s004.zip › X6.jpg]

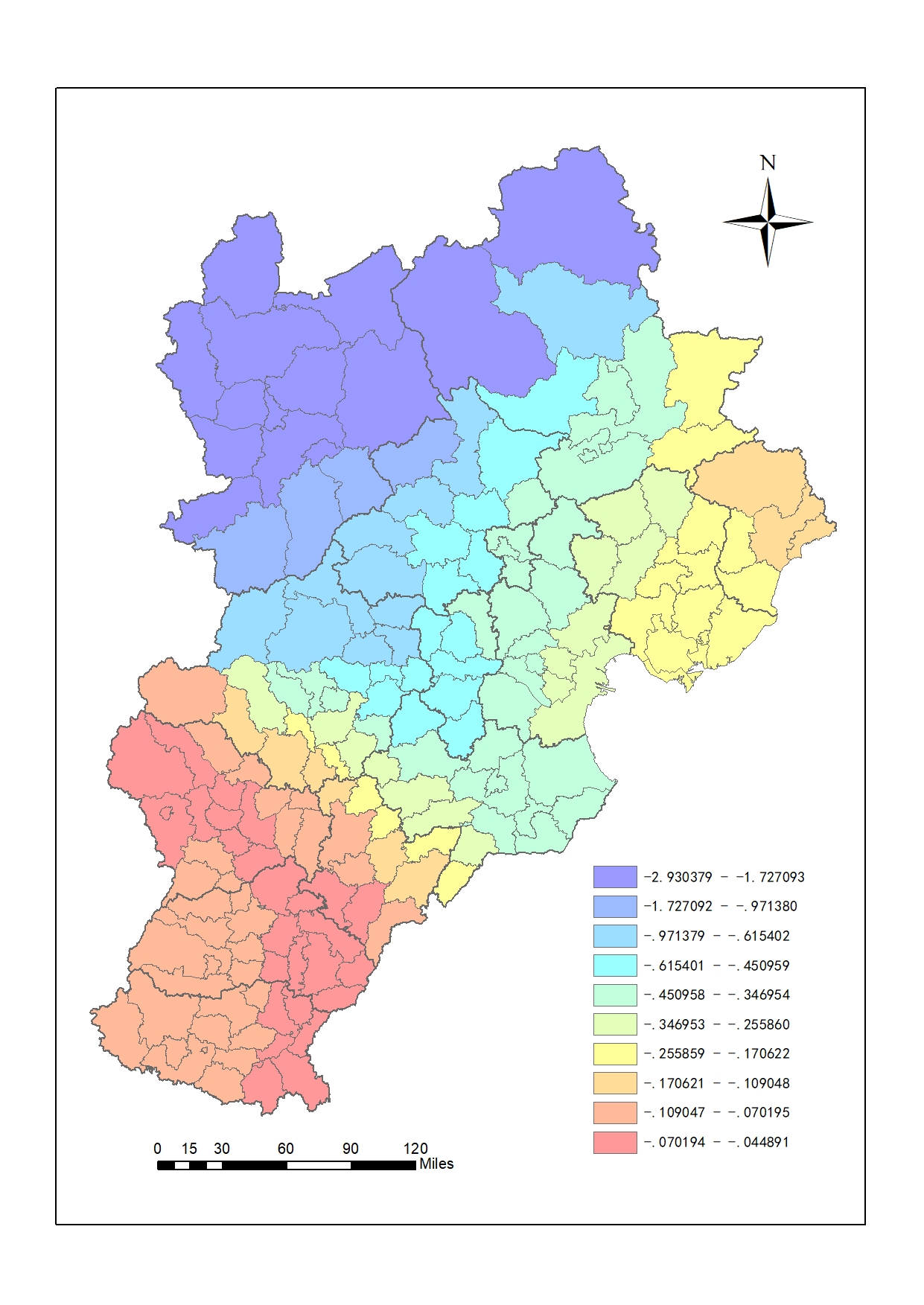

Supplement: S4 Fig — Distribution map of the driving effect of X3, X5, X6, X9 on residential land prices. (ZIP) [file pone.0256710.s004.zip › X9.jpg]

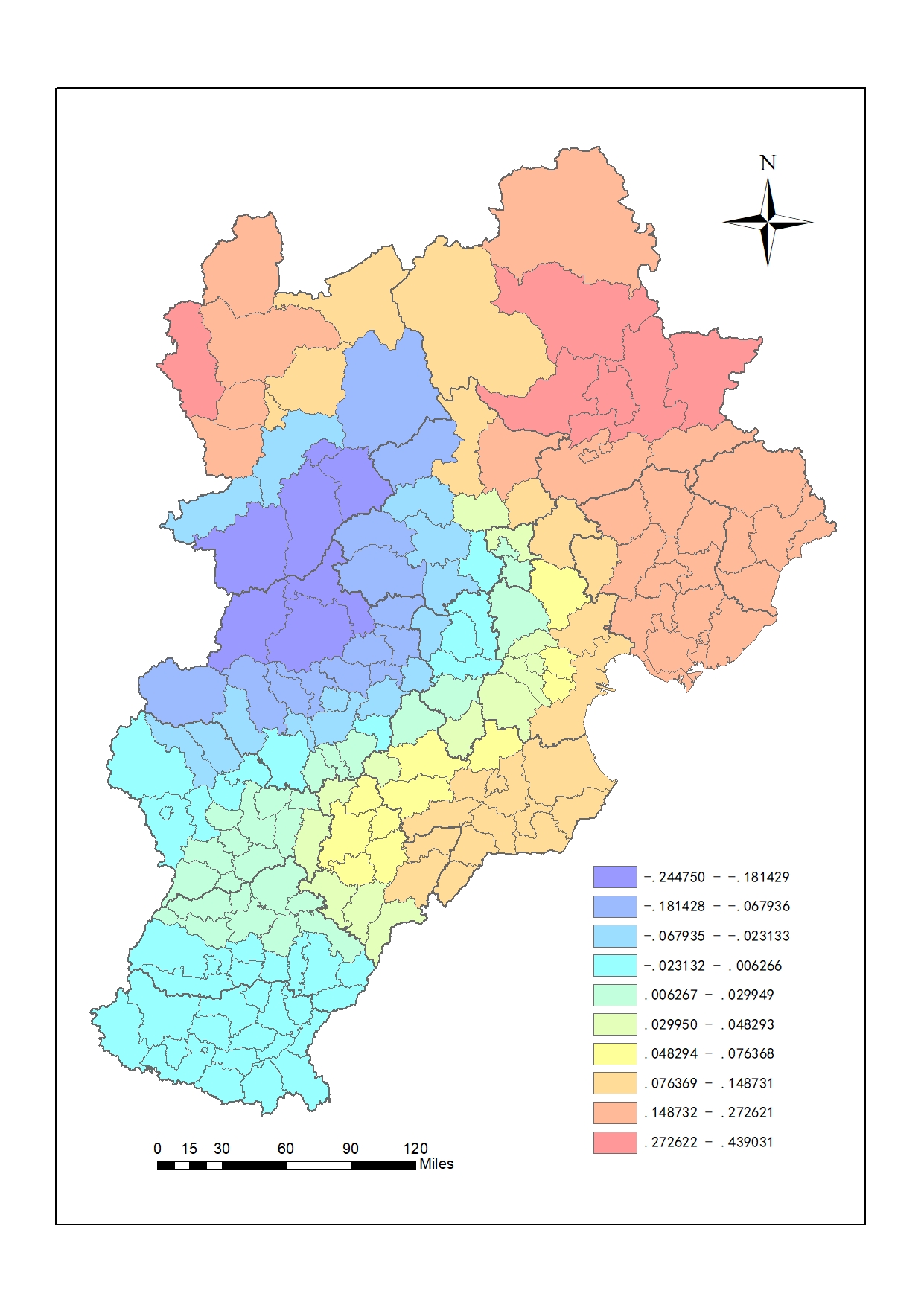

Supplement: S5 Fig — Distribution map of the driving effect of X1, X2, X7 on residential land prices. (ZIP) [file pone.0256710.s005.zip › X1.jpg]

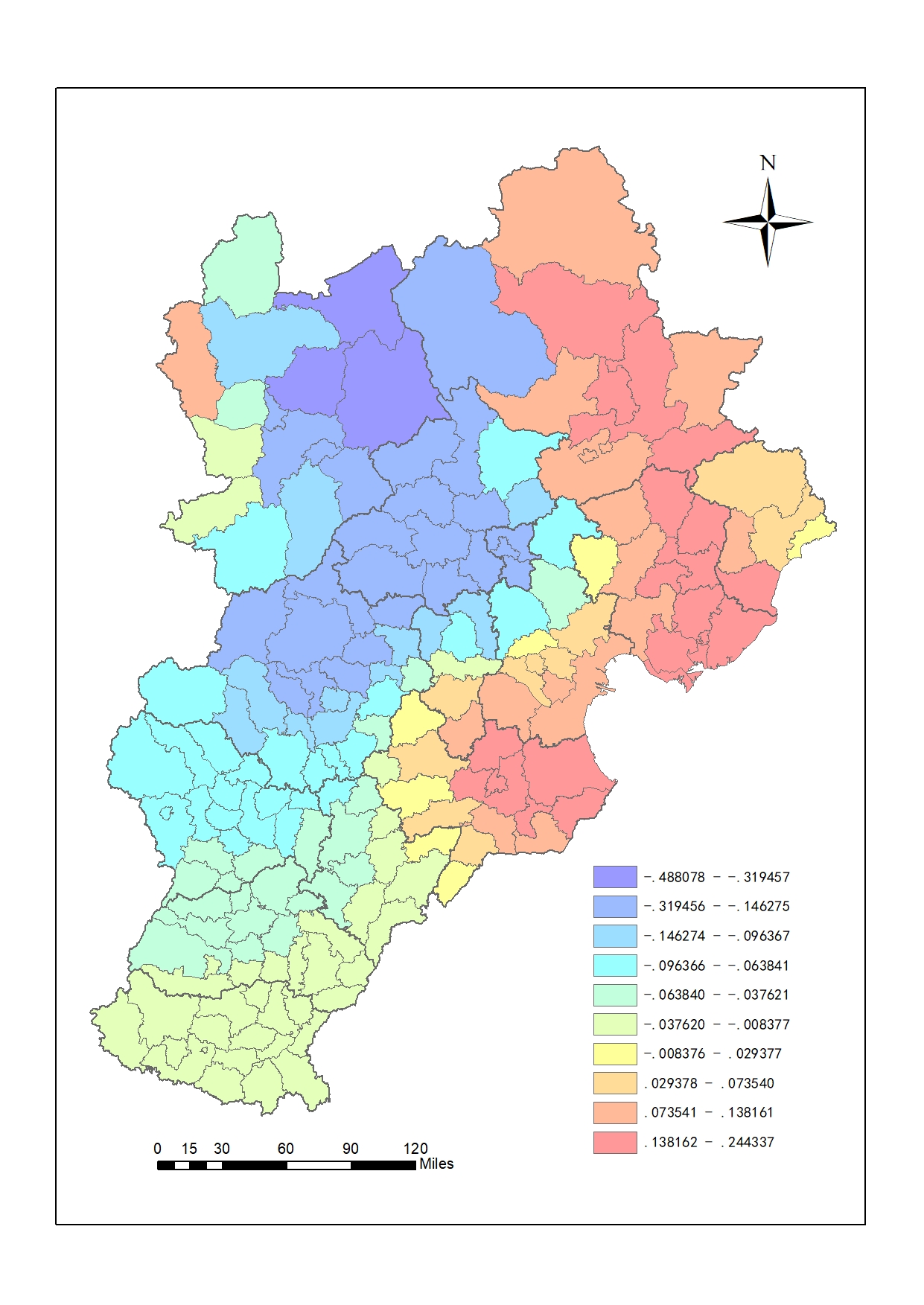

Supplement: S5 Fig — Distribution map of the driving effect of X1, X2, X7 on residential land prices. (ZIP) [file pone.0256710.s005.zip › X2.jpg]

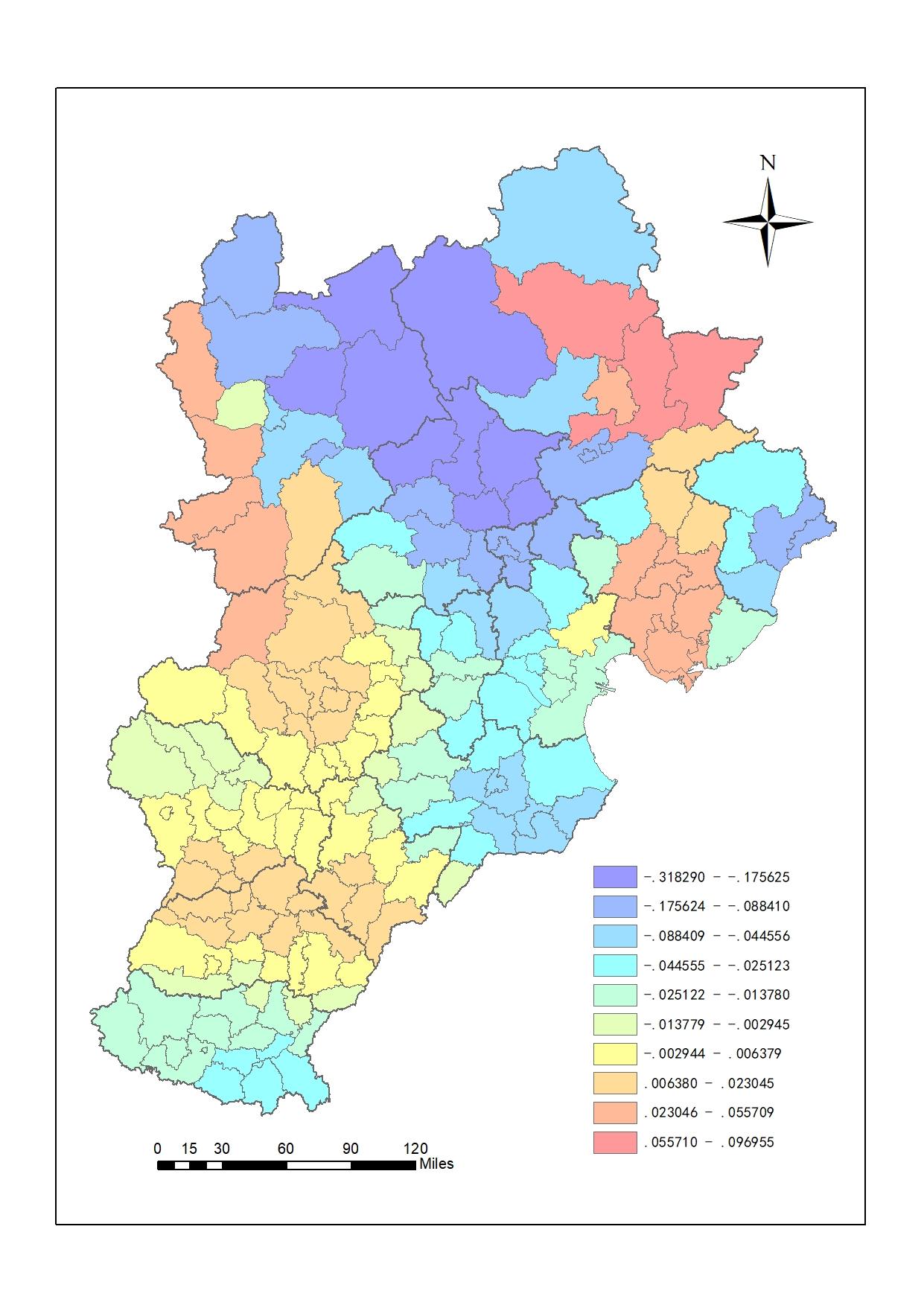

Supplement: S5 Fig — Distribution map of the driving effect of X1, X2, X7 on residential land prices. (ZIP) [file pone.0256710.s005.zip › X7.jpg]
